# Left Atrial Septal Pouch in Cryptogenic Stroke

**DOI:** 10.3389/fneur.2015.00057

**Published:** 2015-03-24

**Authors:** Jonathan M. Wong, Dawn M. Lombardo, Ailin Barseghian, Jashdeep Dhoot, Harkawal S. Hundal, Jonathan Salcedo, Annlia Paganini-Hill, Nathan D. Wong, Mark Fisher

**Affiliations:** ^1^Department of Medicine, California Pacific Medical Center, San Francisco, CA, USA; ^2^Division of Cardiology, Department of Internal Medicine, University of California Irvine, Irvine, CA, USA; ^3^Department of Neurology, University of California Irvine, Irvine, CA, USA; ^4^Departments of Anatomy and Neurobiology, University of California Irvine, Irvine, CA, USA; ^5^Departments of Pathology and Laboratory Medicine, University of California Irvine, Irvine, CA, USA

**Keywords:** cryptogenic stroke, ischemic stroke, transesophageal echocardiography

## Abstract

**Background:** The left atrial septal pouch (LASP), an anatomic variant of the interatrial septum, has uncertain clinical significance. We examined the association between LASP and ischemic stroke subtypes in patients undergoing transesophageal echocardiography (TEE).

**Methods:** We determined the prevalence of LASP among consecutive patients who underwent TEE at our institution. Patients identified with ischemic strokes were further evaluated for stroke subtype using standard and modified criteria from the Trial of Org 10172 in Acute Stroke Treatment (TOAST). We compared the prevalence of LASP in ischemic stroke, cryptogenic stroke, and non-stroke patients using prevalence ratios (PR).

**Results:** The mean age of all 212 patients (including stroke and non-stroke patients) was 57 years. The overall prevalence of LASP was 17% (*n* = 35). Of the 75 patients who were worked-up for stroke at our institution during study period, we classified 31 as cryptogenic using standard TOAST criteria. The prevalence of LASP among cryptogenic stroke patients (using standard and modified TOAST criteria) was increased compared to the prevalence among other ischemic stroke patients (26 vs. 9%, *p* = 0.06; PR = 1.8, 95% CI = 1.1–3.1, and 30 vs. 10%, *p* = 0.04; PR = 2.2, 95% CI = 1.2–4.1, respectively).

**Conclusion:** In this population of relatively young patients, prevalence of LASP was increased in cryptogenic stroke compared to stroke patients of other subtypes. These findings suggest LASP is associated with cryptogenic stroke, which should be verified by future large-scale studies.

## Introduction

Upwards of 40% of ischemic strokes are of unknown etiology and are known as “cryptogenic” (1). The left atrial septal pouch (LASP), an anatomic variant of the atrial septum (2), may be a site of thrombus formation resulting in cardioembolic stroke. Prior case reports describe thrombi along the left atrial septum in the setting of ischemic stroke or transient ischemic attack (TIA) (3–6), and we have previously described LASP in the setting of cryptogenic stroke (7). A case-control study demonstrated no association between LASP and ischemic or cryptogenic stroke (8); however, that study population was limited to patients older than 50 years of age, and mean age of stroke subjects was over 70 years. The primary aim of the present study was to determine if LASP is associated with cryptogenic stroke in a population that includes young subjects.

## Materials and Methods

**Figure 1:**
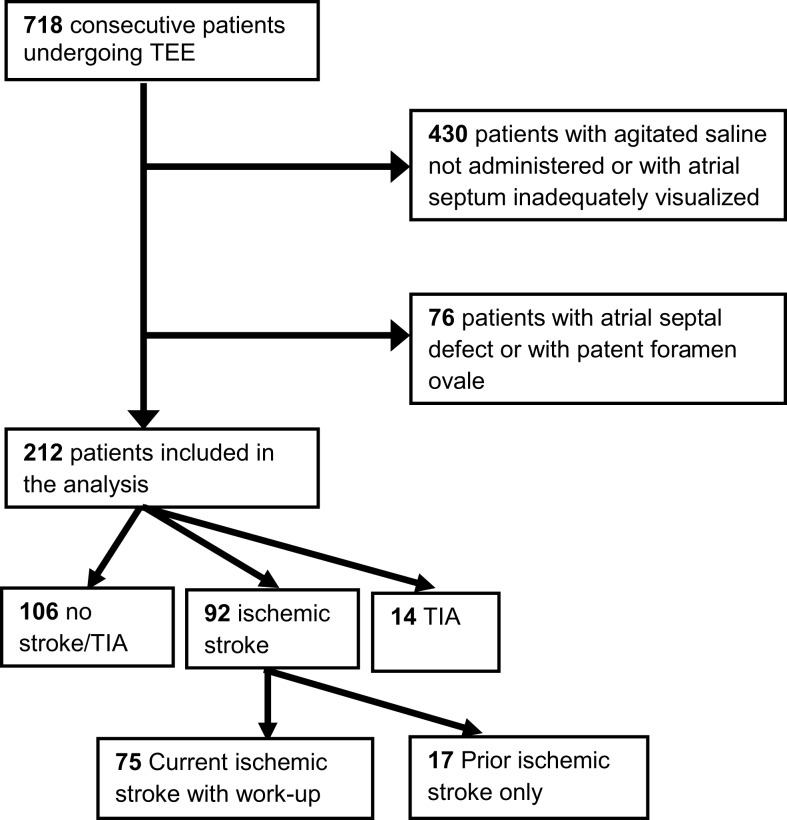
**Flowchart outlining the study exclusion process**. TEE, transesophageal echocardiogram.

In this cross-sectional study, we retrospectively evaluated 718 consecutive patients (with or without stroke) who underwent a transesophageal echocardiogram (TEE) between July 2008 and June 2011 at the University of California, Irvine Medical Center (UCIMC). Patients were excluded from analysis (Figure 1) if the atrial septum was not adequately visualized or if agitated saline injections were not administered (*n* = 430); agitated saline injections with Valsalva maneuver is the standard maneuver for evaluating the presence of patent foramen ovale (PFO) (9, 10). Of the remaining 288 study subjects, we excluded patients with an atrial septal defect (ASD) or PFO (*n* = 76 or 26%) as was done in prior study of LASP and stroke risk (8), because these entities have previously been associated with cryptogenic stroke (11).

We performed a chart review (history, physical exam, consultations, and outpatient notes) for the remaining 212 eligible patients to determine history of hypertension, hyperlipidemia, diabetes mellitus, atrial fibrillation/flutter, ischemic stroke or TIA, coronary artery disease, and congestive heart failure. Of the 92 patients with current or prior ischemic stroke, 75 were worked-up for ischemic stroke at UCIMC during the study period. Stroke was verified by review of magnetic resonance imaging of the brain and vascular imaging (by computed tomography, magnetic resonance, ultrasound, and/or arteriography). These 75 patients were subtyped by one vascular neurologist according to criteria developed for the Trial of Org 10172 in Acute Stroke Treatment (TOAST) (12). Ischemic strokes were classified into one of five categories: large artery atherosclerosis, cardioembolic, lacunar, other determined cause, and cryptogenic. We also used modified TOAST criteria, in which ischemic strokes with multiple competing identified etiologies were excluded from the cryptogenic category. For example, a patient with atrial fibrillation and severe carotid artery stenosis would be excluded from the modified cryptogenic stroke group. This group represents an ischemic stroke population that is without any identifiable etiology by conventional work-up. The neurologist was blinded to the presence of LASP. Inter-observer agreement using TOAST criteria has been studied previously and shown to be reliable (13). The study was approved by the Institutional Review Board of UCIMC.

**Figure 2:**
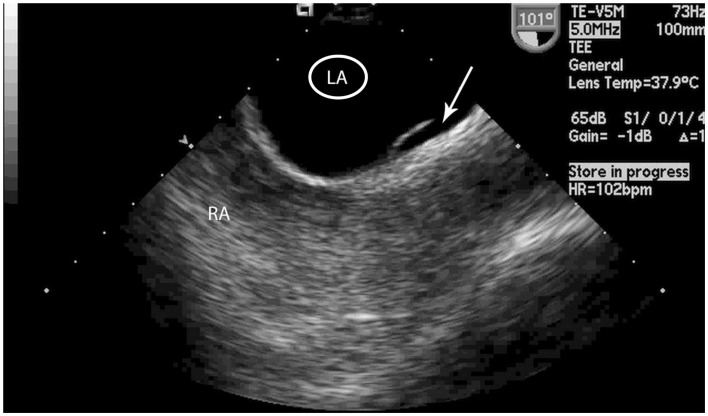
**Transesophageal echocardiogram image of the left atrial septal pouch**. Transeophageal echocardiogram image of the left atrial septal pouch (arrow). LA, left atrium, RA, right atrium.

All TEEs were interpreted by two of four cardiology fellows. The cardiologists were blinded to all patient information, including whether or not the patient experienced a stroke. The interatrial septum was inspected using 2-dimensional echocardiography and one of four anatomical possibilities was recorded: fused septum, LASP, PFO, or ASD. A LASP was defined as fusion at the caudal limit of the zone of overlap between the septum primum and septum secundum, whereby a blind-ending pouch is formed that communicates exclusively with the LA (Figure 2). The presence of a PFO was determined if microbubbles were seen in the LA after agitated saline injections were administered and Valsalva maneuver was performed by the patient. The presence of a PFO was also confirmed by the original echocardiography report. Any differences in interpretation between the cardiology fellows were adjudicated by a third cardiologist and director of the echocardiography laboratory (DL).

### Statistical analysis

We compared differences in stroke risk factors in patients with and without LASP using the Chi-square test of proportions for categorical variables (Fisher’s exact test for 2 × 2 tables) and *t*-tests for continuous variables. The Kappa statistic was used to calculate agreement above chance among the raters of LASP. We determined the LASP prevalence ratio (PR), which is preferable to the odds ratio in cross-sectional studies (14), using two sets of comparison groups: (1) patients without a history of ischemic stroke or TIA (*n* = 106), and (2) patients presenting with non-cryptogenic ischemic strokes (*n* = 44 by TOAST and *n* = 52 by modified TOAST). Multiple logistic regression analysis was performed to see if the effect of LASP on stroke risk was changed by adjusting for age (continuous), gender, and the stroke risk factors of atrial fibrillation/flutter, congestive heart failure, coronary artery disease, diabetes mellitus, ever-smoker, hyperlipidemia, hypertension, and prior history of ischemic stroke. Predictors were included in the final model if the *p* value was <0.15. A *p* value of <0.05 was considered statistically significant for other analyses. All analyses were performed using STATA (version 12.0, StataCorp, College Station, TX, USA).

## Results

The mean age of all 212 subjects (including stroke and non-stroke) was 57 years. The age range for the 92 ischemic stroke patients was 16–90 years and for the 106 non-ischemic stroke patients was 16–86 years. The overall prevalence of LASP was 17% (*n* = 35). The Kappa statistics comparing the agreement between each rater combination ranged from 0.70 to 0.83, indicating good to very good agreement. The distribution of demographic information and ischemic stroke risk factors by the presence of LASP is shown in Table 1. No variable was statistically different between the two groups.

**Table 1:** **Patient characteristics categorized by the presence or absence of LASP**.

Covariate	LASP (*n* = 35)	No LASP (*n* = 177)	*p*-value
**Demographics**
Age, years (mean ± SD)	57 ± 17	57 ± 15	0.78
Male sex (%)	20 (57)	106 (60)	0.85
**Stroke risk factors (%)^a^**
Atrial fibrillation/Flutter (%)	12 (34)	44 (25)	0.30
Congestive heart failure (%)	7 (20)	28 (16)	0.62
Coronary artery disease (%)	7 (20)	34 (20)	1.00
Diabetes mellitus (%)	10 (29)	51 (29)	1.00
Ever-smoker (%)	14 (40)	65 (37)	0.85
Hyperlipidemia (%)	14 (40)	75 (43)	0.85
Hypertension (%)	17 (49)	115 (66)	0.06
Prior ischemic stroke (%)	8 (23)	21 (12)	0.09

*LASP, left atrial septal pouch; SD, standard deviation*.

*^a^Three persons with missing values*.

There were 92 subjects with ischemic stroke, 14 with a TIA history, and 106 without a history of ischemic stroke or TIA (Figure 1). The prevalence of LASP among these subgroups was: 20, 14, and 14%, respectively. Subjects who were worked-up at UCIMC for ischemic stroke during the study period (*n* = 75) were further characterized by stroke subtype using standard TOAST criteria: large artery atherosclerosis = 19, cardioembolic = 8, lacunar = 8, other determined etiology = 9, cryptogenic = 31.

Table 2 shows the prevalence of LASP among patients with no ischemic stroke/TIA, ischemic stroke, and cryptogenic or non-cryptogenic stroke. The prevalence of LASP in cryptogenic stroke patients using standard TOAST criteria was nearly triple that compared with non-cryptogenic ischemic stroke patients (26 vs. 9%, *p* = 0.06; PR = 1.8, 95% CI = 1.1–3.1). This association was greater and statistically significant when using modified TOAST criteria to define cryptogenic stroke (30 vs. 10%, *p* = 0.04; PR = 2.2, 95% CI = 1.2–4.1). The prevalence of LASP among the other stroke subtypes were 5.3% for large artery atherosclerosis (*n* = 1), 13% for cardioembolic (*n* = 1), 0% for lacunar (*n* = 0), and 22% for other determined etiology (*n* = 2).

**Table 2:** **Prevalence of the left atrial septal pouch among ischemic and cryptogenic stroke populations**.

Subgroup	No. LASP/total	LASP prevalence (%)	Prevalence ratio (95% CI)	*p*-value
No stroke/TIA	15/106	14	1.2 (0.9–1.7)	0.31
All ischemic strokes^a^	18/92	20	
**TOAST criteria^b^**
Non-cryptogenic	4/44	9	1.8 (1.1–3.1)	0.06
Cryptogenic	8/31	26	
**Modified TOAST criteria^c^**
Non-cryptogenic	5/52	10	2.2 (1.2–4.1)	0.04
Cryptogenic	7/23	30	

*CI, confidence interval; LASP, left atrial septal pouch; TIA, transient ischemic attack*.

*^a^Includes current and prior ischemic stroke, with subtyping performed for 75 ischemic stroke patients who were worked-up for stroke at UCIMC during the study period*.

*^b^Cryptogenic stroke defined as either (1) no likely etiology despite extensive evaluation or (2) two OR more potential causes*.

*^c^Cryptogenic stroke defined as (1) no likely etiology despite extensive evaluation*.

There was no significant difference in prevalence of atrial fibrillation/flutter in patients with or without LASP (34 vs 25%, *p* = 0.30). No patient with cryptogenic stroke (standard or modified TOAST criteria) had evidence of atrial fibrillation/flutter. In multivariate analyses comparing LASP in cryptogenic stroke patients vs. patients who had no history of stroke/TIA, adjustment for age, gender, and stroke risk factors (see Table 1) did not significantly change the effect size using standard TOAST criteria (OR = 2.3 vs. 2.1) or modified TOAST criteria (OR = 2.3 vs. 2.7).

## Discussion

This study is the first to demonstrate evidence for an association between LASP and cryptogenic stroke. Among 212 patients who underwent TEE at UCIMC, LASP was roughly twice as prevalent among cryptogenic ischemic stroke patients compared to non-cryptogenic stroke patients. Our findings suggest that some individuals are susceptible to cardioembolism from LASP.

*In utero*, the interatrial septum consists of a separated septum primum and septum secundum, forming a conduit known as the foramen ovale (2). A LASP is formed when these septa fuse incompletely, creating a blind-ending pouch that communicates exclusively with the left atrium. The LASP may behave similarly to the left atrial appendage, the site where thrombi most commonly form in patients with non-valvular atrial fibrillation (15). Current mechanistic theories as to why thrombi have been observed within the LASP include intra-pouch stasis and chronic inflammation at the site of the pouch (4). Gurudevan and Krishnan proposed that brisk laminar flow along the interatrial septum protects against thrombus formation in the LASP (4).

Our findings contrast with a case-control study by Tugco and colleagues (8), which found no association between LASP and ischemic or cryptogenic stroke in 187 first-ever ischemic stroke patients (of which 69 were cryptogenic). A few possibilities might explain why our findings are not consistent. First, our study population consisted of hospitalized patients across all age groups referred for TEE who were predominantly Hispanic or Caucasian, whereas the prior study included first-time ischemic stroke patients who were older than 50 years of age and were predominantly Hispanic or African–American (>80%). Since many ischemic stroke patients do not routinely undergo TEE, our study is subjected to selection bias. However, we selected this patient population because it tends to have higher prevalence of cryptogenic strokes. Although our study was not designed to have the power for analysis of subgroups by age, younger patients may be more likely to experience cryptogenic stroke in the setting of LASP. The process of fusion between the septum primum and septum secondum may be dynamic after birth, and the size and extent of blood stasis of the LASP may decrease with age (2). Second, the prevalence of LASP among our control group was smaller, perhaps due to differences in LASP prevalence across different populations or differences in the accuracy of LASP diagnosis. Nonetheless, despite a relatively small statistical power, we detected a statistically different prevalence of LASP among patients with cryptogenic stroke. A more recent report also noted no association of LASP with cryptogenic stroke, although the latter was defined differently and apparently included small deep infarcts/lacunar strokes (16).

There are several study limitations to be considered. The absolute number of cryptogenic stroke subjects and absolute difference in LASP between cryptogenic and non-cryptogenic stroke subjects was relatively small, affecting the study’s statistical power and ability to detect a statistical difference of the prevalence of LASP between some subgroups. Because this was a retrospective cross-sectional study, we could not determine whether LASP increases cryptogenic stroke risk longitudinally or directly mediates cryptogenic stroke. A significant number of patients were excluded because either the atrial septum was not well-visualized or saline contrast injection was not performed, which may have affected LASP prevalence rates in this cohort. It is possible that antiplatelet or anticoagulant use at the time of stroke, which was not captured in our study, differed between patients with and without LASP. Additionally, detection of atrial fibrillation/flutter was made by chart review only; increasing evidence supports subclinical atrial fibrillation as an under-diagnosed risk factor for cryptogenic stroke (17, 18) and therefore it is possible that our cohort had higher rates of undetected atrial fibrillation. Finally, while LASP prevalence rates were increased for cryptogenic stroke patients, prevalence rates were not significantly increased for the entire group of ischemic stroke patients as compared to non-stroke patients.

In conclusion, the LASP is a recently discovered anatomic entity and our findings suggest that its presence is associated with cryptogenic stroke. This should be verified by future large-scale studies. Further research is warranted to understand the pathophysiology of LASP and the conditions which may promote its thrombogenic potential (19).

## Conflict of Interest Statement

The authors declare that the research was conducted in the absence of any commercial or financial relationships that could be construed as a potential conflict of interest.

## Abbreviations

ASD, atrial septal defect; CI, confidence interval; LASP, left atrial septal pouch; PFO, patent foramen ovale; OR, odds ratio; PR, prevalence ratio; TEE, transesophageal echocardiography; TIA, transient ischemic attack; TOAST, Trial of Org 10172 in Acute Stroke Treatment; UCIMC, University of California Irvine Medical Center.
